# Fasciotomy through multiple small skin incisions for the treatment of early acute osteofascial compartment syndrome in children

**DOI:** 10.1186/s13018-020-01742-2

**Published:** 2020-07-17

**Authors:** Xiaowei Yuan, Jun Wu, Xiangyang Qu, Ming Li, Linjun Jiang, Xing Liu

**Affiliations:** Department of Orthopaedics, Children’s Hospital of Chongqing Medical University, Ministry of Education Key Laboratory of Child Development and Disorders, Chongqing Engineering Research Center of Stem Cell Therapy, China International Science and Technology Cooperation base of Child Development and Critical disorders, 136# Zhongshan 2 road, Yuzhong District Chongqing, 400014 People’s Republic of China

**Keywords:** Surgical approach, Decompression, Osteofascial compartment, Scar, Skin grafting

## Abstract

**Background:**

The purpose of the present study is to investigate the therapeutic effect of fasciotomy through multiple small skin incisions for the treatment of early osteofascial compartment syndrome in children.

**Methods:**

From January 2009 to May 2017, 56 pediatric patients with early osteofascial compartment syndrome in their limbs were admitted into our department and treated with multiple small skin incisions for decompression at the early stage. The skin incisions, function, and sensation of the limbs were followed up.

**Results:**

The osteofascial compartment syndrome was diagnosed at 7.4 ± 2.1 h after injury, and then fasciotomy was performed at 1.4 ± 0.4 h later. The average procedure time of fasciotomy was 12.7 ± 4.8 min. No postoperative incision infections or neurovascular injuries were observed in all the patients. The incisions completely healed in 7–10 days with an average healing time of 8 days without secondary suture. The patients were followed up for an average of 5.1 years. No Volkmann’s contractures in the injured limbs were found. The appearance, electromyography, and nerve conduction velocity of the affected limbs were not significantly different from that of the contralateral limbs. All the patients were free of symptoms and were fully recovered of sensation and function, being an “excellent” outcome at the latest follow-up.

**Conclusion:**

Fasciotomy through multiple small skin incisions, which can be useful to decompress the compartment pressure with fewer complications, is a simple and effective strategy for the treatment of early osteofascial compartment syndrome in children.

## Introduction

Osteofascial compartment syndrome (OCS) is one of the serious emergency conditions in pediatric orthopedics that is often accompanied by extremity fractures, vascular injuries, soft tissue crush injuries caused by car accidents or earthquakes, and improper external fixation [1, 2]. It is a progressive acute illness that can lead to total or partial loss of limb sensorimotor functions or even severe organ failure if it is not diagnosed and treated in time. Therefore, it is critical to diagnose and treat this disease at its earliest stage [3, 4].

Surgical decompression is the only effective treatment of this disease if conservative treatment fails [1–3]. Traditional method of decompression was fasciotomy through a large incision, which immediately reduced the compartment pressure to minimize ongoing tissue damage and long-term functional deficit of the myoneural tissues within the compartment, being the standard treatment for OCS. However, there were neurovascular injury, scar, operation trauma, superficial or deep wound infections, and other serious complications in the traditional fasciotomy. Twelve percent to 21% of cases require a second operation such as skin grafting or tissue expansion to close the wound [5, 6]. Since these serious complications, the traditional fasciotomy is applied scrupulously in the early OCS, especially in the doubtful cases, missing the best time for treatment, leading to neuromusrosis and subsequent poor outcomes.

Development of a fasciotomy through little wound is of great value in the early OCS. In 1993, DiStasio et al. used multiple relaxing skin incisions to facilitate the closure of difficult lower extremity wounds when they noted extreme tension (skin blanching, gapping) caused by swelling or soft tissue loss. There was no requirement of soft tissue coverage procedures, and no serious complications such as delayed wound healing, superficial or deep wound infections, and compartment syndrome. The results confirmed that the use of multiple relaxing skin incisions was a safe, simple, and reliable technique for closure of complicated swelling wound [7]. Relaxation incisions were performed to treat compartment syndrome caused by snakebite injury [8, 9]. Akira et al. found that relaxation incisions could be useful not only to wash out the venom from the bitten region but also to decompress subcutaneous and compartment pressure of the hand bitten by the venomous snake “Japanese mamushi” [10].

From January 2009 to May 2017, 56 pediatric patients with early OCS in the extremities were admitted to our department and treated with multiple small skin incisions for decompression. These patients were followed up for an average period of 5.6 years; their records were analyzed to evaluate the clinical effects of fasciotomy through multiple small skin incisions for the treatment of early OCS in children.

## Methods

### Patients

This study was approved and supervised by the Ethics Committee of the Children’s Hospital of Chongqing Medical University and was performed in accordance with the ethical standards laid down in the 1964 Declaration of Helsinki. The early osteofascial compartment syndrome (OCS) was diagnosed and evaluated by two experienced surgeons according to signs and symptoms of the patients [2–4]. Exclusion criteria were patients older than 18 years, with neurovascular injury of the limbs, and incomplete medical records. From January 2009 to May 2017, 56 pediatric patients with early OCS were involved in this study, and their records such as demographic profiles, injury mechanisms, cause of OCS, and hospitalization information were retrieved and analyzed retrospectively.

### Surgical procedure

**Fig. 1:**
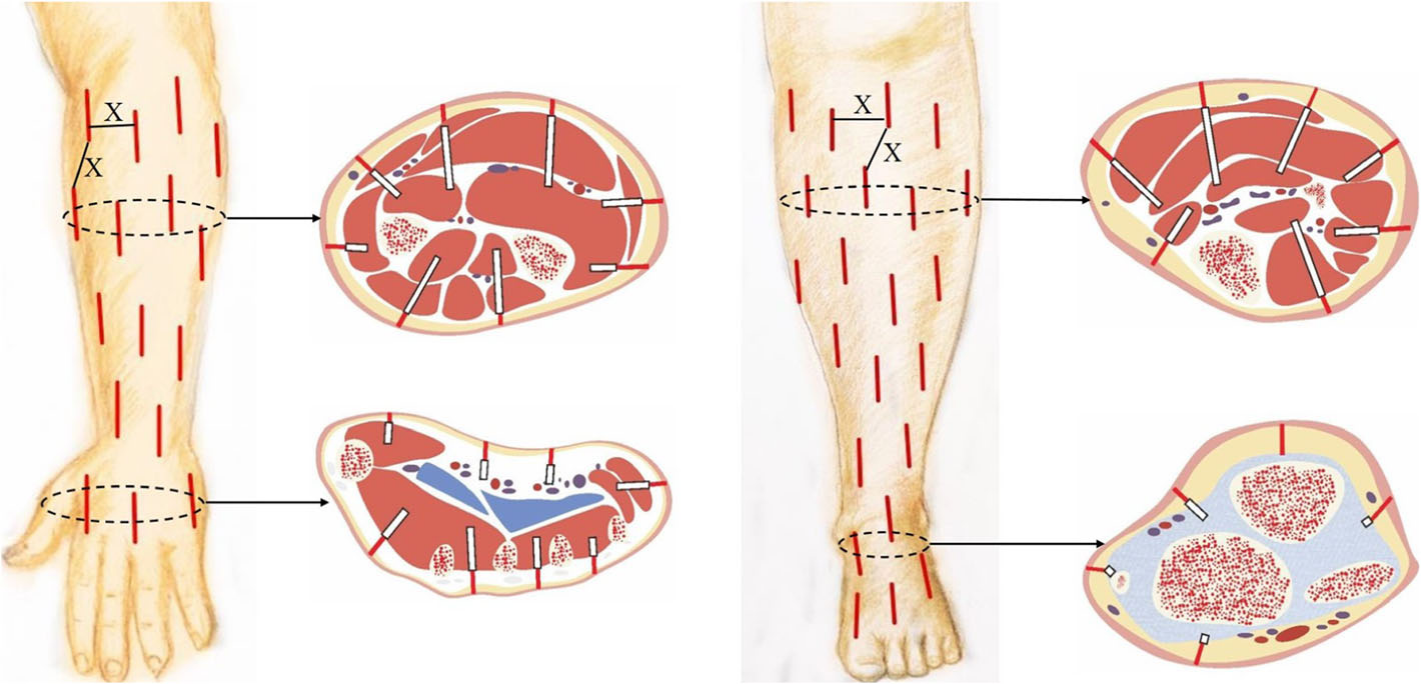
The operation diagram. X, distances between the adjacent skin incisions, about 0.5–1.0 cm; red lines, skin incisions were made with a sharp blade; black box, subcutaneous and deep fascias were separated with a vascular clamp

Once the patient was suspected to suffer from OCS, the external fixation was removed immediately, and the affected limb was raised to relieve swelling. Mannitol (20%) was injected intravenously at 5 ml/kg and repeated once after 2 h. If no improvement was observed after 2–4 h, local reticular incisions were immediately performed on the affected limb for decompression after satisfactory general anesthetization and disinfection. Skin incisions were made from 0.5 to 1.0 cm using sharp blades. Subcutaneous and deep fascias were separated to the intermuscular septa using a vascular clamp until exudates were observed. Muscle bulges were even observed in some cases. Multiple small incisions were made every 0.5–1.0 cm for decompression in the area with obvious swelling and high tension (Fig. 1). To avoid injuries to the blood vessels and nerves, closed reduction and Orthofix external fixation were performed in the patients with radial and ulnar shaft fractures or tibial and fibular shaft fractures. The other fractures were fixed by Kirschner needles. The wound surface was covered with gauze after the surgery, and the affected limb was laid flat.

Dehydration with 20% mannitol and proper use of antibiotics were employed to prevent infection in the incision. The incision dressing was changed regularly. Routine bacterial cultures were performed once or twice at 3 and 7 days after the surgery before the incision healed. Drug sensitivity tests were performed to adjust the antibiotics used.

### Follow-up

The skin incisions, function, and sensation of the limbs were followed up. The clinical outcome at the time of the latest follow-up was recorded and graded as “excellent” (free of symptoms, no loss of sensation or function), “fair” (major recovery of sensation and function, the need for a minor assistive device), or “poor” (major loss of sensation or function, the need for a major assistive device) [11, 12]. At the last follow-up, electromyography and nerve conduction velocity were used to evaluate the neuromuscular function of the affected limbs.

## Results

The demographic profiles of the patients were shown in Table 1. From January 2009 to May 2017, a total of 56 pediatric patients with early osteofascial compartment syndrome (OCS), 39 males and 17 females aged between 1.2 and 13.8 years old with an average of 6.5, were treated with reticular incisions in our department. Among these patients, 14 cases showed the condition in the forearm, 29 cases in the lower leg, 5 cases in the hand, and 8 cases in the foot. Thirty-two cases were injured in car accidents, ten patients suffered from injuries by dropped heavy objects, eleven children fell from a height, and three patients suffered a sports trauma. Fifty-one of the 56 patients were associated with fractures in the injured limbs, including humeral supracondylar fractures, radius and ulna fractures, metacarpal fractures, tibia and fibula fractures, and foot fractures, which were all closed fractures without open wounds. When admitted into the hospital, the patients exhibited marked swelling and increasing pain in the affected limb, 37 of them (66.1%) with apparent passive stretching pain, 25 (44.6%) with tension blisters, 11 (19.6%) with weak arterial pulsation, 18 (32.1%) with cold extremities, and 16 (28.6%) with numbness.

**Table 1:** The demographic profiles of the 56 patients with early osteofascial compartment syndrome

Sex	
Male (*n*)	39
Female (*n*)	17
Age (years)	6.5 (1.2–13.8)
Injury mechanism	
Traffic accidents (*n*)	32
Crushing (*n*)	10
Fall from height (*n*)	11
Sports trauma (*n*)	3
Accompanying fracture (*n* = 51)	
Humeral supracondylar fracture (*n*)	15
Radius and ulna fracture (*n*)	11
Metacarpal fracture (*n*)	9
Tibia and fibula fracture (*n*)	10
Foot fracture (*n*)	6

The OCS was diagnosed at 7.4 ± 2.1 h after injury, and then fasciotomy was performed at 1.4 ± 0.4 h later that was 8.5 ± 2.4 h after injury. Among the 51 children with limb fractures, 40 cases were firstly treated by fasciotomy to reduce the pressure of the involved osteofascial compartments, and reduction of the fractures was performed when the swelling of the limbs was effectively relieved. But fracture reduction and fasciotomy were performed in the same operation for the other 11 cases whose limb swelling was relatively mild and pressure of the involved compartments could be quickly relieved after the fasciotomy. All the fasciotomy was performed through reticular incisions. The average procedure time of fasciotomy (from multiple incisions started to decompression completed) was 12.7 ± 4.8 min, and the amount of bleeding was 18.7 ± 4.1 ml.

No postoperative incision infections or neurovascular injuries were observed in all the patients. No bacterial growth was observed in the bacterial cultures. The incisions completely healed in 7–10 days with an average healing time of 8 days without secondary suture. No patients required a secondary surgery for decompression through traditional fasciotomy via a large incision. No osteomyelitis or bone delayed union was found in the patients. The mean hospital stay was 8.3 days (range from 7 to 10 days).

**Fig. 2:**
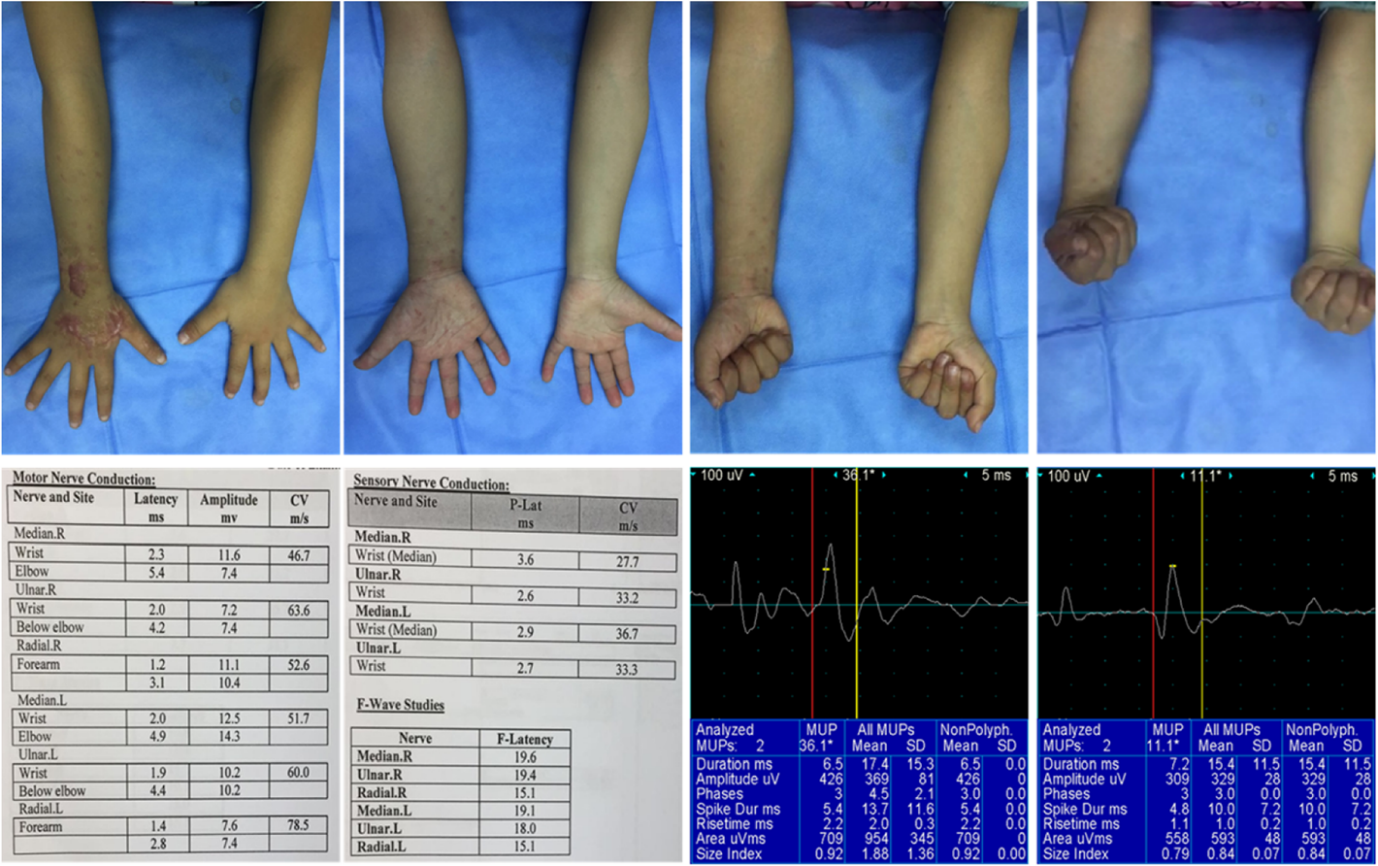
A 5-year-old girl presented to our department with a painful, swollen right hand. The physical examination revealed edema in the right hand and forearm. Active and passive motion of the hand was limited by pain. Capillary refilling was delayed, and the radial pulse was undetectable. Emergency fasciotomy through multiple small skin incisions was performed at about 7 h after injury. The incisions completely healed in 9 days without infections or secondary suture. At the latest follow-up, that was 5 years after surgery, the girl was free of symptoms and was fully recovered of sensation and function, being an “excellent” outcome. The electromyography and nerve conduction velocity of the affected limbs were not significantly different from that of the contralateral limbs

The patients were followed up for 1–8.5 years after surgery with an average of 5.6 years. No Volkmann’s contractures in the injured limbs were found. At the last follow-up, the appearance, electromyography, and nerve conduction velocity of the affected limbs were not significantly different from that of the contralateral limbs. All the patients were free of symptoms and were fully recovered of sensation and function, being an “excellent” outcome at the latest follow-up. There was a total satisfactory rate of 98.2%. A typical case is shown in Fig. 2.

## Discussion

Osteofascial compartment syndrome (OCS) is a serious emergency condition in pediatric orthopedics. If not treated properly, it is liable to lead to ischemic muscle contracture (Volkmann’s contracture) with a high rate of disability. OCS not only affects the body functions but also significantly influences the psychological health of pediatric patients [1–4]. Due to impacts from heavy objects, crush injuries, or long-term compression (overtight or prolonged fixation by small splints or plaster casts), the removal of the pressure causes blood reperfusion and results in the bleeding and reactive swelling of injured tissues (mainly muscular tissues), which leads to the increased volume of contents in intermuscular septa followed by increased pressures and acute OCS. Acute OCS can cause rhabdomyolysis and acute muscular atrophy, disseminated intravascular coagulation, electrolyte disturbance, renal failure, and other complications and even death in some severe cases.

The typical symptoms and physical signs of OCS can usually be described clinically as the “5P” symptoms: namely, pain or from pain to painless, pallor, pulselessness, paralysis, and paresthesia. However, when the injured limbs show the typical “5P” symptoms, the best time for treatment has often been missed, which may result in serious consequences such as physical disability and even amputation [13]. Severe pain not consistent with the degree of injuries, passive stretching pains in the limbs, and sensory loss within the region where the injured nerves are distributed have long been considered as important clinical signs indicating OCS. In the present study, OCS was diagnosed based on the presence of evident post-traumatic swelling and pain, especially passive stretching pain. In other clinical retrospective studies, it has been found that 100% of the subjects suffered passive stretching pains in the limbs and 60% experienced paresthesia [14]. OCS can be diagnosed if the osteofascial compartment pressure is higher than 30 mmHg in patients with a history of trauma and a high degree of local swelling in the limbs [13, 15]. However, pediatric patients often have poor descriptive capability, do not cooperate during physical examination, and cannot accurately express their sensation. In addition, pressure measuring devices are not equipped in the hospitals in underdeveloped areas, and pressure measurement is inaccurate in some cases, leading to limited application of pressure measurements in the diagnosis of OCS [16]. Therefore, health care providers should carefully observe the conditions of the patients and be vigilant for the occurrence of this disease [3, 13, 17].

Even though conservative treatments, such as mannitol, can be applied for this disease at an early stage, the only timely and effective treatment for this disease is effective decompression therapy prior to the aggravation of the condition when the ischemic changes in the muscular tissues are still reversible [1–3]. Currently, the complete incision for osteofascial compartment decompression at an early stage is the only way to effectively prevent ischemic necrosis of the muscles and nerves. Incision decompression should be performed immediately once the diagnosis is confirmed and short-term conservative treatments are not effective. Early incision is preferred when compared with delayed treatment [18, 19]. The gold standards for the treatment of OCS are early diagnosis and emergent surgical decompression through fasciotomy within 8 h. Therefore, the treatment for OCS is focused on early decompression but is not unlimited conservative treatment [20, 21]. In the present study, incision decompression was immediately performed for all the patients who did not show improvement 2–4 h after conservative therapy, which produced significant therapeutic effects.

The choice of surgical incision size has been controversial. Large incisions are traditionally considered to be more conducive to the removal of necrotic tissue and complete decompression. However, large incisions are prone to complications of infection and prolonged periods of dressing changes. Moreover, second-phase wound sutures, even dermoplasty, are required for wound healing, which leaves visible scars that affect the patient’s appearance [5, 6]. Thus, we prefer multiple small incisions for decompression. Incision decompression was performed in the areas with marked swelling and elevated tension, which on one hand provides complete and effective decompression and on the other hand allows for closed reduction and external fixation of fractures and avoids secondary surgeries. In the present study, the results suggested that fasciotomy via multiple small skin incisions was simple and fast operation procedure with less trauma, shorter operation time, without space restrictions, and was suitable for emergency treatment and surgeries in remote mountainous areas and field sites. Moreover, multiple small skin incisions are associated with less intraoperative blood loss, good postoperative recovery of local skin, little impact on the appearance, easier second-phase wound healing, and low infection rate.

However, small incision has its limitations. The multiple small skin incisions can only provide timely and effective decompression for osteofascial compartment, but cannot completely clear the ischemic necrotic tissues and repair the injured blood vessels or nerves. Therefore, fasciotomy via multiple small skin incisions is only recommended for early OCS without neurovascular injury.

## Conclusion

Acute osteofascial compartment syndrome is a serious emergency condition in pediatric orthopedics which requires an immediate fasciotomy to reduce the compartment pressure to minimize ongoing tissue damage and long-term functional deficit of the myoneural tissues within the compartment. The present study shows fasciotomy through multiple small skin incisions, which can be useful to decompress the compartment pressure with fewer complications, and is a simple and effective strategy for the treatment of early acute osteofascial compartment syndrome in children.

## Data Availability

The datasets analyzed in the study are available from the corresponding author on reasonable request.

## Abbreviation

OCS: Osteofascial compartment syndrome
